# Characterization of antibiotic-resistance traits in *Akkermansia muciniphila* strains of human origin

**DOI:** 10.1038/s41598-022-23980-6

**Published:** 2022-11-12

**Authors:** Rossella Filardi, Giorgio Gargari, Diego Mora, Stefania Arioli

**Affiliations:** Department of Food Environmental and Nutritional Sciences (DeFENS), University of Milan, Via Celoria 2, 20133 Milan, Italy

**Keywords:** Antimicrobial resistance, Microbiology, Bacterial genomics

## Abstract

*Akkermansia muciniphila*, a commensal bacterium commonly found in healthy gut microbiota, is widely considered a next-generation beneficial bacterium candidate to improve metabolic and inflammatory disorders. Recently the EFSA’s Panel on Nutrition, Novel food, and Food Allergens has declared that pasteurized *A. muciniphila* DSM 22959^T^ (also Muc^T^, ATCC BAA-835) can be considered safe as a novel food, opening the door to its commercialization as a food supplement. Despite its recognized health benefits, there is still little information regarding the antimicrobial susceptibility of this species and reference cut-off values to distinguish strains with intrinsic or acquired resistance from susceptible strains. In this study, we combined a genomic approach with the evaluation of the antibiotic susceptibility in five human *A. muciniphila* isolates. Genomic mining for antimicrobial resistance genes and MICs determinations revealed that only one strain harboring *tetW* gene showed resistance to tetracycline, whereas all *A. muciniphila* strains showed low sensitivity to ciprofloxacin and aminoglycosides with no genotypic correlation. Although all strains harbor the gene *adeF*, encoding for a subunit of the resistance-nodulation-cell division efflux pump system, potentially involved in ciprofloxacin resistance, the susceptibility towards ciprofloxacin determined in presence of efflux pump inhibitors was not affected. Overall, our outcomes revealed the importance to extend the antibiotic susceptibility test to a larger number of new isolates of *A. muciniphila* to better assess the safety aspects of this species.

## Introduction

*Akkermansia muciniphila* is a Gram-negative anaerobic bacterium abundantly present in the human intestine where it uses mucin as carbon and nitrogen source^1,2^. Several studies demonstrated that this species is beneficial to host health and its abundance correlated inversely with people suffering from metabolic syndrome (e.g.: obesity, diabetes, cardiometabolic disease) and inflammatory bowel disease^3–5^. In particular, the type strain, *A. muciniphila* DSM 22959^T^ (also Muc^T^, ATCC BAA-835), has been extensively studied and is considered a next-generation beneficial bacterium due to its protective effect against obesity and metabolic disorders^6–8^. The first intervention study targeting overweight/obese insulin-resistant humans showed that supplementation with *A. muciniphila* DSM 22959^T^ is safe, well-tolerated and improves metabolic parameters^9^. Nevertheless, the use of *A. muciniphila* in food or pharmaceutical formulations depends on the demonstration of its efficacy and safety within regulatory frameworks. In 2020, EFSA's Panel on Biological Hazards (BIOHAZ) did not recommend this species for the Qualified Presumption of Safety (QPS) list due to safety concerns^10^. Although they recognized the safe and well-tolerated administration of live or pasteurized cells of *A. muciniphila* DSM 22959^T^ in humans and mice, they stressed the possible involvement of the ability to degrade mucin with pathological processes and a possible association of this species with neurological diseases. Moreover, EFSA’s panel highlighted the presence of several antimicrobial resistance genes (ARGs) in the genomes of this species. *A. muciniphila* together with other anaerobic gut commensals associated with human health (*Bacteroides* spp., *Clostridium butyricum*, *Faecalibacterium prausnitzii*) are gaining significant attention for their potential application in food supplements and pharmaceutical formulations, however these genera and microbial species do not have a history of safe use yet^11,12^. An important aspect to consider, when evaluating their safety, is the potential to carry ARGs that could be acquired from harmful bacteria. The gut microbiome, the main source of these health-promoting microbes, is considered a reservoir of ARGs and the anaerobic commensal species represent the main contributors to the human intestinal resistome^13,14^. Specifically, *A. muciniphila* is thought to be particularly plastic and prone to gaining antimicrobial resistance (AMR)^15,16^. In Europe, information on AMR for bacteria deliberately introduced into the food chain is of paramount importance to declaring a microorganism safe for human and animal consumption. For this purpose, phenotypic testing based on the determination of a minimum inhibitory concentration (MIC) for a selected group of antimicrobials, together with the complementary search of the whole genome sequences for the presence of known ARGs, should be performed^17^. Nevertheless, while genome mining for ARGs is routinely performed, standardized methods for the MIC evaluation have not yet been defined for this microorganisms. Furthermore, there is a lack of specific microbiological cut-off values to be used to distinguish strains with intrinsic or acquired resistance from susceptible strains^18,19^, mainly due to the limited number of available cultivable strains. Recently, EFSA Panel on Nutrition, Novel food, and Food Allergens (NDA) expressed a positive opinion on the safety of pasteurized *A. muciniphila*, opening the door for its use in food supplements and in food for special medical purposes^20^. With the introduction of *A. muciniphila* in the food chain*,* the evaluation of the antimicrobial susceptibility of this bacterium becomes fundamental to meet the safety recommendations of EFSA. To the best of our knowledge, only few studies have focused on the antibiotic resistance profile of *A. muciniphila* strains through phenotypic tests^21–23^, while most of the available information derives from genome data analysis^15,23–26^. Furthermore, as most of the phenotypic studies concern the type strain of this species, the antimicrobial susceptibility variation in *A. muciniphila* strains remains largely unexplored. As such, the aim of the present study is to characterize the AMR profile of novel human isolated strains of *A. muciniphila* including the type strain DSM 22959^T^, by integrating phenotypic and in silico approaches, to provide new insight into the safety of this promising species.

## Results and discussion

### Comparative genome sequence analyses

**Figure 1:**
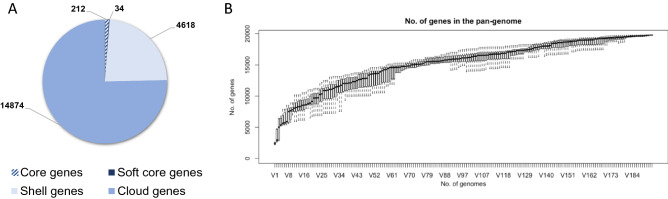
Comparative genomics analysis of the 193 *Akkermansia* sp. genomes. (**A**) Pangenome content. (**B**) New genes content variation as new genomes are added to the analysis.

**Figure 2:**
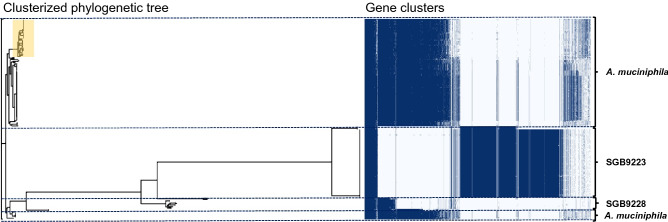
Phylogenetic tree of the 193 *Akkermansia* sp. genomes. The shell genes were used for the clusterization based on gene presence (dark blue) or absence (white). The genomes are clusterized into three groups corresponding to *Akkermansia* sp. SGB9228 (11 genomes), SGB9223(69 genomes), *A. muciniphila* (113 genomes). The yellow zone of the tree highlights the position of the newly isolated *A. muciniphila* strains characterized in this study.

In our study, we isolated *A. muciniphila* strains from feces of healthy volunteers. Species-specific qPCR assay^2^ along with the analysis of the melt curve allowed to select fecal samples positive for the presence of *A. muciniphila* to be used for a further enrichment step and isolation procedure. Although 9 donors out of 16 (= 56%) tested positive, a total of 10 isolates of *A. muciniphila* were obtained from only 5 subjects, highlighting that difficulties in culturing *A. muciniphila* from fecal samples could limit the isolation and characterization of new strains^25,27^. The *A. muciniphila* isolates, preliminarily identified via partial 16S rRNA gene sequencing, were differentiated into 5 subtypes, different from the type strain, by molecular typing (Supplementary Table S1 and Figure S1). Each subtype belonged to a different donor, indicating that different subjects could be colonized by different strains. Then, one isolate for each subtype was selected for whole-genome sequencing. General genomic features of the *A. muciniphila* genomes are summarized in Table 1 and in Supplementary Figure S1. De novo assembly of the genomic data, after contigs cleaning (cut-off 500 bp), revealed genome sizes ranging from 2.65 to 3.42 Mbp according to previous observations (average: 3.01 Mbp; median: 2.97 Mbp, completeness of assemblies 98%, contaminations detected ranged from 1.02 to 9.77%)^15^. The G + C contents of the genomes ranged from 54.9 to 55.8%. The presence of a high value of marker genes duplicated in strain Amap1 justifies the high number of very small contigs and consequently the extra length of the genome. However, this does not lead to a significant increase in terms of the number of unique predicted genes. Previous large-scale genomic-based analyses^15,26^, focusing on *Akkermansia* spp diversity, have shown that *A. muciniphila* is not the only species of the genus *Akkermansia.* These studies highlighted the presence of at least 4 new candidate species, which are characterized by remarkable whole-genome divergence despite similar 16S rRNA gene sequences. Therefore, the 16S rRNA analysis alone is not sufficiently discriminatory for the identification of the *Akkermansia* spp.. To unequivocally identify the new strains at the species level, the whole-genome Average Nucleotide Identity (ANI) values were calculated using fastANI between the newly sequenced genomes and the *A. muciniphila* type strain genome^28^. Compared to the wild type, all five strains exhibited ANI scores ranging between 97.4 and 99% and were therefore confirmed as *A. muciniphila* according to the ANI species limit definition^29^. We also performed a pan-genome analysis on the 5 isolates, along with 188 publicly available *Akkermansia* genomes (NCBI). The comparative genomic analysis of the newly isolated and publicly available (n = 188) *Akkermansia* genomes identified a pan-genome of 19,738 genes, including 212 core genes (shared between the 99 and 100% of the strains), 34 soft-core genes (between 95 and 99%), 4618 shell genes (between 15 and 94%), and 14,874 cloud genes (less than 15% of the strains) (Fig. 1). The large pan-genome and the small core genes show great genetic diversity within *Akkermansia* spp. The coding sequence alignment of the 193 genomes performed for the pan-genome analyses, with the Roary matrix built on the presence-absence of shell genes, highlighted 3 clusters (Fig. 2). According to Karcher et al.^26^, two clusters referred to the new putative species, namely SGB9228 and SGB9223, the third to *A. muciniphila* species. The analysis revealed that the new five strains were all identified as *A. muciniphila* species.

**Table 1:** General genomic features of *A. muciniphila* strains.

*A. muciniphila* strain	Genome size (Mb)	GC content (%)	N° of contigs	N° of predicted genes (unique)	N° of features	CDS	RNAs	rRNA	N50 (bp)	L50	Completeness (%)	Contamination (%)
DSM 22959^T^	2.66	55.8	1	2098	2632	2570	62	9	2,664,295	1	98	0
Sap1	2.87	55.2	63	2273	2961	2893	68	9	436,824	3	98	1.02
Amap1	3.42	54.9	363	2750	3770	3707	68	9	122,068	9	98	9.77
Vtp7	3.16	55.1	233	2521	3394	3327	67	9	424,687	3	98	2.95
Rcp22	2.96	55.3	87	2341	3014	2958	60	7*	219,669	4	98	0
Amup9	2.97	55.8	190	2410	3157	3102	58	7*	178,662	5	98	0

*CDS* Coding sequences.

*The discrepancy between the number of rRNA genes in strains Rcp22 and Amup9 compared to the reference DSM 22959^T^ and the other strains is likely due to the draft genome sequence. Specifically, two 16S rRNA genes and two 5S rRNA genes are missing in strains Amup9 and Rcp22 strains, respectively.

### Antimicrobial resistance genes and mobile DNA elements

EFSA-FEEDAP guidelines encourage the use of whole-genome sequences to characterize new candidate bacterial strains regarding their potential functional traits of concern, such as the presence of genes encoding for, or contributing to resistance to antimicrobials relevant to their use in humans and animals^17^. To investigate the presence of ARGs in *A. muciniphila* strains, a homology-based search was performed using Resistance Gene Identifier (RGI) on Comprehensive Antibiotic Resistance Database (CARD)^30^. To date, most of the available information on the antibiotic resistance profile of *A. muciniphila* derives from genome data analysis^15,23–26^. When Van Passel and colleagues^24^ analyzed the genome of *A. muciniphila* DSM 22959^T^, they found two potential β-lactamase genes and one gene coding for a 5-nitroimidazole antibiotic resistance protein, commonly found in other intestinal anaerobic bacteria*.* However, Gòmez-Gallego^3^ reported that the recent inspection of the genome annotation of *A. muciniphila* DSM 22959^T^ did not reveal any ARGs of concern. This was further confirmed by Machado et al.^23^ in a recent publication focused on the antimicrobial resistance profile of *A. muciniphila* DSM 22959^T^. According to the previous studies^15,26^, the most frequently ARGs found in *A. muciniphila* genomes are *adeF*, *aph(6)-Id, sul2, InuC* and *aph(3″)-Ib*. ARGs found in the genomes of the new *A. muciniphila* stains are listed in Table 2. The gene *aph(6)-Id*, associated with resistance to aminoglycosides, involved in streptomycin inactivation^31^, and the gene *sul2*, one of the most prevalent determinants of sulfonamide resistance^32^, were specifically found in the genomes of *A. muciniphila* Vtp7 and Amap1. The latter also carries the *tetW* gene, which confers resistance to tetracycline^33^. All strains were also characterized by the presence of the gene *adeF*. This gene encodes a membrane fusion protein belonging to the resistance-nodulation-cell division (RND) efflux pump system AdeFGH^34^, potentially involved in resistance to fluoroquinolones and tetracycline. However, according to our findings, the *A. muciniphila* strains characterized in this work, harbor only the *adeF* gene, while the other components of the AdeFGH operon were not detected, thus limiting its potential activity in fluoroquinolones—and tetracycline-resistance mechanism. Nevertheless, it is important to underline that Karcher et al.^26^, in their study on genomic diversity and ecology of human-associated *Akkermansia* species, found the gene *adeF* consistently in most of the *A. muciniphila* genomes, but never present in the other *Akkermansia* spp. genomes.

**Table 2:** Identification of ARGs in the genome of *A. muciniphila* strains, according to CARD database ^30^.

Strains	ARG	ARGs family	Resistance mechanism	Drug class	RGI*criteria	Identity of matching region (%)	Length of reference sequence (%)
DSM 22959^T^	*adeF* ^34^	Resistance-nodulation-cell division (RND) antibiotic efflux pump	Antibiotic efflux	Fluoroquinolone; tetracycline	Strict	41.27	99.72
Sap1	*adeF* ^34^	Resistance-nodulation-cell division (RND) antibiotic efflux pump	Antibiotic efflux	Fluoroquinolone; tetracycline	Strict	41.27	99.72
Amap1	*adeF* ^34^	Resistance-nodulation-cell division (RND) antibiotic efflux pump	Antibiotic efflux	Fluoroquinolone; tetracycline	Strict	41.45	99.72
	*tetW* ^33^	Tetracycline-resistant ribosomal protection protein	Antibiotic target protection	Tetracycline	Perfect	100	100
	*sul2* ^32^	Sulfonamide resistant sul	Antibiotic target replacement	Sulfonamide	Perfect	100	100
	*aph(6)-Id* ^31^	APH(6)	Antibiotic inactivation	Aminoglycoside	Strict	99.64	100
Vtp7	*adeF* ^34^	Resistance-nodulation-cell division (RND) antibiotic efflux pump	Antibiotic efflux	Fluoroquinolone; tetracycline	Strict	41.45	99.72
	*sul2* ^32^	Sulfonamide resistant sul	Antibiotic target replacement	Sulfonamide	Perfect	100	100
	*aph(6)-Id* ^31^	APH(6)	Antibiotic inactivation	Aminoglycoside	Strict	99.64	100
Rcp22	*adeF* ^34^	Resistance-nodulation-cell division (RND) antibiotic efflux pump	Antibiotic efflux	Fluoroquinolone; tetracycline	Strict	41.45	99.72
Amup9	*adeF* ^34^	Resistance-nodulation-cell division (RND) antibiotic efflux pump	Antibiotic efflux	Fluoroquinolone; tetracycline	Strict	41.45	99.72

*RGI Resistance Gene Identifier.

ARGs harbored in microbial commensals can become a significant hazard if transferred and acquired by pathogens, considering that the gut provides ideal conditions for gene exchange being an environment rich in microbes^14^. According to Guo et al.^15^ *A. muciniphila* acquired genes during its evolution, from a wide range of taxa associated with human intestinal habitat, through horizontal gene transfer. Furthermore, de Nies et al.^16^ highlighted that *A. muciniphila* has a plastic genome, that is particularly prone to acquire ARGs under antibiotics selective pressure. In light of the above, we wanted also to screen the *A. muciniphila* strains for the presence of mobile genetic elements (MGEs), possibly involved in the ARGs transfer. The only identified trait of concern is related to the Amap1 strains, which has a putative transposon (Tn6205) in its genome associated to the AGRs *aph(6)-Id* and *sul2*, thus posing risks of gene transfer events^35^. One insertion sequence (IS), ISAmu1, has been identified in the genomes of *A. muciniphila* Sap1 and Amap1 as well as in *A. muciniphila* DSM 22959^T^. In general, ISs can move ARGs as part of a composite or compound transposon, that is a region bounded by two copies of the same IS that can move as a single unit. However, there are examples of a single IS mobilizing an adjacent region that may contain one or more ARGs. ISs can also affect antibiotic resistance by driving the expression of the adjacent genes^36^. However, in the newly isolated *A. muciniphila* strains the ISs sequences identified are not flanked by ARGs. Plasmids, which could be involved in ARGs mobilization, were found in none of the *A. muciniphila* strains (Supplementary Figure S1).

### Antibiotic sensitivity

To fully address the antibiotic resistance profile of the *A. muciniphila* strains, we performed a phenotypic test based on MIC determination for a selected group of antibiotics. According to the EFSA guidelines^17^, for Gram-negative bacteria the antibiotics tested should be those for *Enterobacteriaceae*. For antibiotic susceptibility testing, the culture medium used must allow the growth of the strains under assessment and not interfere with antibiotics^17^. IsoSensitest (IST) is the nutrient medium recommended by the British Society for Antimicrobial Chemotherapy. However, for specific bacteria, other formulations may be required^17,37^. In our study, we used a modified version of IST (sIST) to allow the growth of *A. muciniphila*. Therefore, we first verified whether the additional medium ingredients could interfere with antibiotic sensitivity. To this aim, we used *E. coli* Nissle as a reference strain of the *Enterobacteriaceae* family. MICs determined in sIST were compared with those obtained in cation-adjusted Mueller Hinton Broth, a conventional susceptibility test medium^38^. MICs determined in the two media agreed (Table 3), as such, we can state that the sIST components did not interfere with the antibiotic sensitivity assay. Overall, the MICs obtained for the antimicrobials tested are similar between the new *A. muciniphila* isolates and the type strain, showing a similar level of sensitivity within the species (Table 3). All strains were sensitive to ampicillin, tetracycline, colistin, fosfomycin, and sulfamethoxazole. Only the strain Amap1 showed resistance towards tetracycline as expected due to the presence of *tetW* gene in its genome. Strains Amap1 and Vtp7, which were genotypically predicted to be resistant to sulfonamides, due to the presence of gene *sul2*, resulted sensitive to sulfamethoxazole comparably to all other strains. Indeed, the presence of ARGs does not always translate into a resistant phenotype, as already reported for bacterial isolates with silent ARGs^39^. The latter underlines the importance of combining genotypic and phenotypic tests to better define antimicrobials resistance. All *A. muciniphila* strains, including the type strain, showed low sensitivity to ciprofloxacin and aminoglycosides (gentamicin, kanamycin, streptomycin). Comparing the phenotypic susceptibilities with the genotypic AMR profiles, all strains harbor a gene potentially involved in ciprofloxacin resistance mediated by antimicrobial efflux (*adeF*). Concerning the low sensitivity to aminoglycosides, only the Amap1 and Vtp7 strains have a genetic determinant presumably involved in streptomycin resistance (*aph (6) -Id*)^31^, indicating that poor sensitivity to this class of antibiotics in all the strains tested could be due to a more general intrinsic mechanism. Indeed, intrinsic resistance to aminoglycoside is common in anaerobic bacteria^40,41^. Furthermore, it is known that some Gram-negative bacilli are resistant to aminoglycoside due to a transport defect or change in outer membrane permeability and this mechanism causes cross-reactivity to all aminoglycosides^42,43^. According to EFSA recommendations, the MIC values obtained should be compared with public data on the specific species under study. To the best of our knowledge, few published studies have addressed the antibiotic susceptibility profile of *A. muciniphila* through phenotypic tests^21–23^. Moreover, these studies tested different molecules using different methodologies, making difficult compare data. Dubourg et al.^21^ has highlighted the increased colonization of the human intestinal microbiota by the *Verrucomicrobia* phylum following a broad-spectrum antibiotic regimen. In this context, they evaluated the sensitivity of *A. muciniphila* DSM 22959^T^ to different antibiotics, using the E-test method on Wilkins-Chalgren agar plates with 5% blood. They found that the type strain is susceptible to imipenem, piperacillin/tazobactam, and doxycycline, and resistant to vancomycin, metronidazole and penicillin G. Later, Cozzolino et al.^22^ tested the susceptibility of *A. muciniphila* DSM 22959^T^ to antimicrobials recommended for *Lacticaseibacillus rhamnosus*, using the E-test strips on Brain Heart Infusion agar plate. The MIC values they obtained for ampicillin, tetracycline, and streptomycin (respectively 2, 0.75, 128 mg L^−1^) are in line with those obtained in this study, indeed, the MIC values for gentamicin and kanamycin (respectively 4, 12 mg L^−1^) were consistently lower compared to our data, so that they categorized this strain as susceptible to these aminoglycosides. This discrepancy can be partially explained by the different methods used for phenotypic testing (broth dilution in our work vs. E-test method in Cozzolino et al.^22^) or by the growth medium used (sIST in our work *vs* BHI Cozzolino et al.^22^). Disagreements caused by methodologies used for classifying microbial species into resistant or susceptible phenotype are present in the literature^19,44^. However, it is important to highlight that broth dilution method is widely accepted for phenotypic tests based on MIC determination. Recently, Machado et al.^23^ evaluated the sensitivity of *A. muciniphila* DSM 22959^ T^ strain to the antimicrobials using a mucin-supplemented Pepton Yeast Glucose and comparing two methods (broth microdilution *vs* E-test method). Their results showed that DSM 22959^T^ is resistant to gentamicin, kanamycin, streptomycin, and ciprofloxacin, consistently with our findings. Furthermore, we found our results in line with the EFSA 2021 document^20^ and Ouwerkerk et al.^45^, where authors reported that *A. muciniphila* BAA-835 (= DSM 22959^T^) presented high resistance levels to aminoglycosides, and ciprofloxacin similarly to other *A. muciniphila* strains.

**Table 3:** MICs (mg l^−1^) of tested antibiotics for *A. muciniphila* strains.

Strain	AMP	GEN	KAN	STR	TET	COL	FOSF	SULF	CIPR	CIPR + CCCP	CIPR + PaβN
*Enterobacteriaceae* cut-off values^19^	8	2	8	16	8	2	8	256	0.06	–	–
DSM 22959^ T^	4	256	512	256	< 2	< 2	< 4	< 4	128–256	128	64–128
Sap1	4	256	512	256	< 2	< 2	4	< 4	128	128	128
Amap1	4	128	512	512	64	< 2	4	< 4	128	128	128
Vtp7	4	128	512	256	< 2	< 2	< 4	< 4	64–128	64	64
Rcp22	4	256	512	512	< 2	< 2	< 4	< 4	128–256	128	128
Amup9	2	128	512	256	< 2	< 2	< 4	< 4	128	64	64–128
*E. coli* Nissle 1917 (sIST)	8	2	8	16	8	0.5	64	32	< 0.25	nd	nd
*E. coli* Nissle 1917 (caMHB)	4–8	2	4–8	8	4	0.5	32	16	< 0.25	nd	nd

*AMP* Ampicillin; *GEN* Gentamicin; *KAN* Kanamycin; *STR* Streptomycin; *TET* Tetracycline; *COL* Colistin; *FOSF* Fosfomycin; *SULF* Sulfamethoxazole; *CIPR* Ciprofloxacin; *CCCP* carbonyl cyanide 3-chlorophenylhydrazone; *PaβN*, phenylalanine-arginine beta-naphthylamide; *sIST* supplemented Iso-Sensitest broth, *CaMHB* cation adjusted Muller Hilton broth; *nd* not determined.

### Investigation of the drug efflux as resistance mechanism towards ciprofloxacin

**Figure 3:**
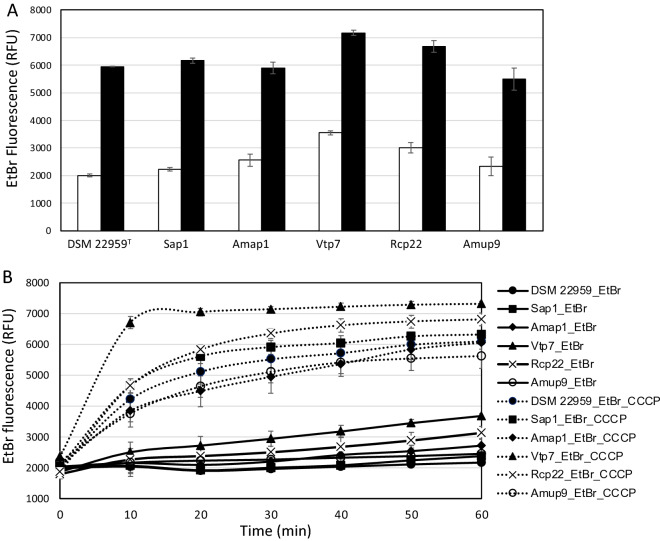
(**A**) Accumulation of ethidium bromide in *A. muciniphila* strains in absence (white bars) and in presence (black bars) of CCCP after 60 min of incubation. (**B**) Kinetics of ethidium bromide accumulation by *A. muciniphila* strains in absence (solid lines) and in presence (dashed lines) of CCCP. Data are shown as the average values of three replicates, with standard deviation. RFU, Relative Fluorescence Unit.

Ciprofloxacin is a fluoroquinolone antibiotic effective against Gram-negative and Gram-positive aerobic bacteria but with low potency against many anaerobic bacteria^46^. Reduced susceptibility to ciprofloxacin may be mediated by (1) alterations in the drug efflux; (2) mutations in the genes coding for drug targets (DNA gyrase and topoisomerase) or (3) change in outer membrane and/or porin permeability to the drug^43,46^. All *A. muciniphila* strains tested here showed scarce sensitivity to ciprofloxacin. Interestingly, we found that all the genomes harbored the gene *adeF*, encoding a membrane protein of a RND drug efflux complex putatively involved in fluoroquinolones resistance. According to previous observations^26^, this ARG is one of the most frequently found in the *A. muciniphila* genomes, therefore understanding its role within this species might be worthy of attention. To confirm the presence of active efflux pumps in the *A. muciniphila* strains, we measured the accumulation of ethidium bromide, a molecule known to be substrate of efflux pumps, in presence and absence of the efflux pump inhibitor (EPI) carbonyl cyanide 3-chlorophenylhydrazone (CCCP). CCCP is a protonophore cell membrane uncoupler that increases membrane permeability to protons, leading to a disruption of membrane potential, thus being a strong inhibitor of RND efflux pumps in Gram-negative bacteria^47^. The assumption of this test is that the higher is the concentration of substrate accumulated inside the bacterial cell, the lower is the efflux level and vice versa. The results showed that the intracellular accumulation of ethidium bromide is significantly (*p* < 0.01, T-test) increased with the addition of CCCP in all the strains, both in terms of total amount and kinetic of ethidium bromide accumulation (Fig. 3). This indirectly confirms that the lowest level of ethidium bromide accumulation, in the absence of CCCP, is determined by active efflux pumps in the *A. muciniphila* strains (Fig. 3). To investigate the involvement of efflux pumps in ciprofloxacin reduced susceptibility, the MICs were determined in absence and in presence of the EPIs CCCP and phenylalanine-arginine beta-naphthylamide (PaβN). PAβN is another broad-spectrum efflux pump inhibitor, capable of significantly reducing resistance to fluoroquinolones in *P. aeruginosa*^48^. It acts as a competitive inhibitor, preventing efflux of the antibiotics by binding the substrate-binding pocket of the efflux pumps and impairing the antibiotic bond to its affinity site^48^. In presence of efflux pump inhibitors, the MICs for ciprofloxacin were equal to or twofold lower than the MIC values determined without EPIs (Table 3). These slight variations in MICs represent the normal standard deviation of MIC dilution tests^49^. Since the addition of the EPIs did not result in a susceptible phenotype, it was excluded the active involvement of efflux pumps in the *A. muciniphila* reduced sensitivity to ciprofloxacin. Since genome analysis revealed no mutations in genes encoding ciprofloxacin targets, the low sensitivity to this molecule could be determined by its poor permeability to the outer membrane as reported for other Gram-negative^43^.

## Conclusions

Our study provides new insights on the phenotypic and genotypic antibiotic resistance profile of *A. muciniphila*, showing a similar level of susceptibility among strains within this species. Between the antibiotics tested, all strains showed poor sensitivity to the fluoroquinolone ciprofloxacin and aminoglycosides, as expected for anaerobic bacteria^40,42,43^. The low sensitivity to these classes of antimicrobials, being widespread among all strains regardless of the presence of ARGs, appears to be caused by intrinsic mechanisms, such as change in the outer membrane and/or porin permeability to the drugs^43^. An ARG related to streptomycin inactivation (*aph (6)-Id*) was found in only two strains, with no effect on the antibiotic sensitivity compared to the other strains. We also investigated the involvement of efflux pump activity in reduced susceptibility to ciprofloxacin, since the *adeF* gene, which encodes for a component of the RND efflux pump system (AdeFGH), was found in all genomes. According to our results, no evidence of the role of an active drug efflux system related to ciprofloxacin reduced susceptibility was observed. However, the involvement of an active efflux against toxic compounds, such as ethidium bromide, was confirmed for all the strains. The ARG *sul2* detected in two strains did not determine a resistant phenotype. Finally, only one of the strains showed traits of concern, harboring three ARGs, one conferring resistance to tetracycline (*tetW*) and two (*aph (6)-Id*, *sul2*) associated with an MGE (Tn6205).

The results of our study underline the urgent need for adequate microbiological breakpoints and standardized protocols to assess the antimicrobial susceptibility of *A. muciniphila* and to favor fair comparative analysis between different laboratories and institutes. Moreover, further studies involving a larger number of *A. muciniphila* strains are necessary to demonstrate the safety of this microbial species.

## Materials and methods

### Isolation of *A. muciniphila* strains

Informed consent was obtained from all subjects of the study. Ethical approval was not required for this study under local legislation and institutional requirements of the University of Milan. All methods were carried out in accordance with proper guidelines of the University of Milan which refer to the WHO Laboratory of Biosafety Manual. For the isolation of new *A. muciniphila* strains, fresh fecal samples were collected from 16 healthy adult human donors, aged 26 to 50 years (34 ± 8 years old) being part of this research group. To proceed with the isolation only with *A. muciniphila* positive-fecal sample, bacterial DNA was isolated from stool samples using DNeasy PowerLyzer PowerSoil Kit (QIAGEN), following the manufacturer’s instructions. Then, the molecular detection of *A. muciniphila* was carried out using species-specific real-time PCR analysis targeted to the variable regions of the 16S rRNA gene sequence of *A. muciniphila*^2^. Real-time PCR was performed using 50 ng of template DNA in a final reaction volume of 15 μl, using the EvaGreen™ kit (Bio-Rad) and following the manufacturer’s recommendations. PCRs were performed in duplicate on a CFX96 instrument (Bio-Rad). Data were recorded as threshold cycles (Ct) and analyzed using Bio-Rad CFX Manager™ software. Fecal samples positive for the presence of *A. muciniphila* (Ct < 30) were used for bacterial isolation following the method of Derrien et al. (2004). Briefly, serial dilutions of positive stool samples were inoculated in a mucin-containing medium for an enrichment step, before spread-plating on the same medium containing 1% (v/v) agar. Plates were incubated at 37 °C for 6 days in an anaerobic chamber (N_2_:H_2_:CO_2_ 90:5:5). Colonies with different morphologies were picked up and grown in Brain Heart Infusion broth (Merck), supplemented with 0.5 g l^−1^ of L-cysteine hydrochloride and 0.25% (w/v) of mucin from porcine stomach (Merck). Isolation of DNA from bacterial cultures was performed using DNeasy UltraClean Microbial Kit (QIAGEN), following the manufacturer’s instructions. Isolates were identified as *A. muciniphila* by use of species-specific PCR^2^. All strains were also verified by partial 16S rRNA gene sequencing. The amplification was done using the universal primers P0 and P6^50^. Finally, the new isolates of *A. muciniphila* were compared by BoxA1 PCR analysis according to van Belkum and Hermans^51^.

### Bacterial strains and culture conditions

*A. muciniphila* DSM 22959^T^ (also ATCC BAA-835; type strain) has been purchased by DSMZ (Deutsche Sammlung von Mikroorganismen und Zellkulturen, Germany). All strains of *A. muciniphila* were grown at 37 °C in an anaerobic chamber (N_2_:H_2_:CO_2_ 90:5:5) for 48 h in yeast medium broth (YM). YM contained (l^−1^): 0.45 g KH_2_PO_4_; 0.45 g K_2_HPO_4_; 0.9 g (NH_4_)_2_SO_4_; 0.9 g NaCl; 0.1 g MgSO_4_; 0.1 g CaCl_2_; 4 g NaHCO_3_; 1.9 ml CH_3_COOH (35 mM); 1 g L-Cysteine HCl monohydrate; 2.5 g yeast peptone; 5 g yeast extract and 2.5 g glucose and, it has been further supplemented with 6 g L-threonine and 2.8 g N-acetyl-D-glucosamine^52^. Antibiotics sensitivity was determined using Iso-Sensitest broth (Oxoid) supplemented with (l^−1^): 0.5 g L-Cysteine HCl monohydrate, 0.01 g Hemin, 0.01 g Vitamin K1, 6 g L-threonine and 2.8 g N-acetyl-D-glucosamine, which were filter-sterilized and added to the autoclaved medium. *Escherichia coli* Nissle 1917 (serotype O6:K5:H1) was isolated from the probiotic product ECN (Cadigroup Farmaceutici) in Luria–Bertani (LB) agar (1,5%, w/v) plates. For the determination of the minimum inhibitory concentrations (MICs) of selected antimicrobials, *E. coli* Nissle 1917 was cultivated overnight in cation-adjusted Muller-Hilton broth^38^ or sIST broth, under aerobic conditions at 37 °C.

### Whole-genome sequencing and genomic analysis of *A. muciniphila* strains

The Whole-genome sequencing of the five *A. muciniphila* strains (Sap1, AmaP1, Vtp7, Rcp22, Amup9), made by Illumina NovaSeq 6000 platform, produced a total of 76′452′151 paired-end reads (Sap1: 10′542′938; Amap1: 10′766′503; Vtp7: 10′530′303; Rcp2: 26′681′187; Amup9: 17′931′220;) long 150 nt with GC content of 55%. The De novo assembling was performed by the assembly toolkit SPAdes 3.14.1^53^. The software fastANI^28^ was used to compare by alignment-free computation of whole-genome Average Nucleotide Identity, the similarity between our draft genomes with 188 *Akkermansia* sp. complete genomes available on http://cmprod1.cibio.unitn.it/akkermansia_genomes/fna/. FASTQ data have been deposited in the European Nucleotide Archive (ENA) of the European Bioinformatics Institute under accession code PRJEB54610. The assembly has been upgraded by SPAdes using the genetic nearest complete genome found with fastANI as reference genome. The CheckM software was used for assessing the quality (completeness and contamination) of the assemblies^54^. The estimation of completeness and contamination is derived from collocated sets of genes that are ubiquitous and single-copy within a phylogenetic lineage. The fraction of marker genes that occur as duplicates is used to calculate the "Contamination" percentage. Instead, the "Completeness" value is obtained by the proportion of the missing markers to the total number of markers used. The number of CDS, RNAs and rRNA were determined using RAST tool kit^55^ associated with BAsic Rapid Ribosomal RNA Predictor (Barrnap) version 0.9 (https://github.com/tseemann/barrnap). The pangenome analyses was performed using Roary^56^ pan-genome pipeline which takes annotated assemblies in GFF3 format produced by Prokka^57^. Resistance Gene Identifier (RGI) on Comprehensive Antibiotic Resistance Database (CARD)^30^, updated April 2022, was used to predict the resistome of our strains based on homology and SNP models. RGI uses Prodigal, a protein-coding gene predictor for prokaryotic genome^58^. To include partial gene prediction, the Prodigal algorithm for small contigs (contigs < 20 kbp) was applied. “Perfect” and “Strict” algorithms were used to detect both perfect match, or previously unknown AMR genes variants, using detection models with CARD's curated similarity cut-offs to ensure the detected variant is likely a functional AMR gene. The Mobile Element Finder Tool^35^ was used to predict mobile genetic elements in our genomes by aligning the assembled contiguous sequences to reference sequences of previously known elements. The database used by Mobile Element Finder tool is the version 1.0.2 updated June 2020.

### Plasmid DNA extraction and analysis

To verify the presence of plasmids in the isolated strains, extrachromosomal plasmid DNA was purified and subjected to agarose gel electrophoresis according to the method of Anderson and McKay^59^. *Lactobacillus helveticus* ATCC 15009, harboring three plasmids, was used as a positive control for plasmid DNA extraction^60^.

### Total cell count and viability assessment by flow cytometry

Total cell counting and viability assessment of the bacterial cultures were performed by flow cytometry (FC), following the ISO 19344 procedure (2015)^61^, with some modifications. Briefly, bacterial samples were diluted (approximately 1 × 10^6^ cells ml^−1^) in filtered phosphate-buffered saline (PBS) pH 7.4 to maintain an events rate in the flow lower than 2000 events s^−1^. The samples were stained with 0.1 µM SYTO™ 24 (Thermo Scientific) and 0.2 µM propidium iodide (PI; Sigma) and incubated in the dark at 37 °C for 15 min. FC analysis was performed with a C6 Plus flow cytometer (BD Biosciences, Milan Italy) with thresholds FSC-H 1000 and SSC-H 1000. All parameters were collected as logarithmic signals. Green (SYTO™ 24) and red (PI) fluorescence were detected in the FL1 (excitation 488 nm, emission filter 530/30) and FL3 (excitation 488 nm, emission filter 670 LP) channels, respectively. Electronic gates on the SYTO24/PI density plot were used to select and measure the total bacterial concentration (events ml^−1^), active fluorescent unit (AFU), and non-active fluorescent unit (nAFu), as described in ISO 19344 (2015)^61^.

### Antimicrobial agents and MICs determination

According to EFSA-FEEDAP document 2018^17^, the antibiotics included in the analysis were those tested for *Enterobacteriaceae* and other Gram-negative bacteria: ampicillin, gentamicin, kanamycin, streptomycin, tetracycline, ciprofloxacin, colistin, fosfomycin, and sulfamethoxazole (Merck). MICs for the antimicrobials listed above were determined by the standard macrodilution broth method^38^ in 24-wells plates, using serial two-fold dilutions of antimicrobials with a bacterial inoculum density of 5 × 10^5^ AFU ml^−1^. To standardize the bacterial density in the inoculum suspensions, cell counting was performed by FC as described above. A bacterial suspension containing 1 × 10^8^ AFU ml^−1^ was prepared by diluting an early stationary phase broth culture. The suspension was then diluted at 1:100 (1 × 10^6^ AFU ml^−1^) in the suitable medium and 1 ml of this dilution was added as inoculum in a final volume of 2 ml. The growth controls contained only inoculated broth without antimicrobials, negative controls comprised media that was not inoculated. When ethanol and dimethylsulfoxide (DMSO) were used respectively as the diluents of tetracycline and sulfamethoxazole, their effect on cell growth was checked. The highest concentrations of ethanol and DMSO used were respectively: 0.63% (v/v) and 0.25% (v/v). In both cases, no inhibition of bacterial growth was seen. Incubation took place in an anaerobic chamber (N_2_:H_2_:CO_2_ 90:5:5) at 37 °C for 48 h. *E. coli* Nissle was cultivated in aerobic conditions for 24 h at 37 °C. The MIC is the lowest concentration of antimicrobial where no visible growth is measured in the wells. MICs were determined at least in duplicates.

### Ciprofloxacin susceptibility assay and efflux pumps inhibitors activity

Susceptibility of *A. muciniphila* strains to ciprofloxacin was also evaluated in the presence of two efflux pump inhibitors (EPIs), carbonyl cyanide 3-chlorophenylhydrazone (CCCP; Merck) or phenylalanine-arginine beta-naphthylamide (PaβN; Merck), to determine the contribution of efflux pumps to ciprofloxacin sensitivity. The concentrations of the EPIs used (CCCP 80 µM; PaβN 10 µM) was decided based on the determination of their MICs. A lower concentration of CCCP (40 µM) was used only for the strains Rcp22 and Amup9, while a concentration of 5 µM of PaβN was used for *A. muciniphila* DSM 22959^ T^ and Amup9. CCCP was soluble in the growth medium with DMSO (1% v/v). DMSO alone was used as a control, and it did not affect cells growth. MICs were determined as mentioned above.

The efflux pumps activity in *A. muciniphila* strains was indirectly assessed by measuring the accumulation of ethidium bromide in the presence and absence of the efflux pump inhibitor CCCP^62^. Briefly, the bacterial strains were cultured in sIST as previously described. The cells were washed in PBS (pH 7.4) and diluted in the same buffer to reach a concentration of 1 × 10^6^ AFU ml^−1^. Two sets of samples were prepared for each bacterial strain. In the first set only ethidium bromide was added at the final concentration of 10 µM. In the second set a sub-inhibitory concentration of CCCP (80 µM and 40 µM) was added and samples were incubated at 37 °C for 10 min. After the incubation, ethidium bromide was added (10 µM). Accumulation of ethidium bromide in cells was measured immediately and recorded every 10 min for 1 h by FC. FC analysis was performed with a C6 Plus flow cytometer (BD Biosciences, Milan Italy) with thresholds FSC-H 1000 and SSC-H 1000. All parameters were collected as logarithmic signals. Ethidium bromide fluorescence was detected in the FL3 channel (excitation 488 nm, emission filter 670 LP). Electronic gates on density plot of the ethidium bromide fluorescence against forward scatter (FSC-H) were used to select and measure the fluorescence intensity to determine the amount of ethidium bromide inside the cells. Indeed, ethidium bromide is a DNA-intercalating agent that fluoresces when bound to DNA. Therefore, fluorescence is higher when intracellular. Samples with an equal concentration of cells but without ethidium bromide were used as blanks and their fluorescence was subtracted from the other measurements.

## Supplementary Information

Supplementary Information.

## Data Availability

The raw reads are deposited in the European Nucleotide Archive (ENA) of the European Bioinformatics Institute under accession code: PRJEB54610.
