# Supplementary material for: Characterization of antibiotic-resistance traits in Akkermansia muciniphila strains of human origin
Source: Sci Rep. 2022 Nov 12;12:19426. doi: 10.1038/s41598-022-23980-6 (PMC9653403; doi:10.1038/s41598-022-23980-6)
Supplement: Supplementary file 1 — Supplementary Information. [file 41598_2022_23980_MOESM1_ESM.pdf]

SUPPLEMENTAL MATERIALS

Characterization of antibiotic-resistance traits in *Akkermansia muciniphila* strains of human origin

Rossella Filardi, Giorgio Gargari, Diego Mora* and Stefania Arioli

Department of Food Environmental and Nutritional Sciences (DeFENS), University of Milan, Italy

E-mail addresses: {rossella.filardi} {giorgio.gargari} {diego.mora} {stefania.arioli}@unimi.it

*Corresponding author

Table S1 Healthy human volunteers, *A. muciniphila* strains, and genotypic characterization. page 2

Figure S1. BoxA1 RAPD profiles of *A. muciniphila* strains. page 3

Figure S2 *A. muciniphila* genomes contig size visualization. page 4

Figure S3 Agarose gel electrophoresis of plasmid DNA extraction from *A. muciniphila* strains. page 5

Full-length image of gels used in Figure S1 page 6

Full-length image of gels used in Figure S3 page 7

References page 8

Table S1. Healthy human volunteers, *A. muciniphila* strains, and genotypic characterization

N°	Subject code	Sex	Age (years)	Ct	*A. muciniphila* strains	16S rRNA sequence identity	BoxA1 RAPD Subtype (ST)
1	RF	F	30	≥ 30.00	-	-	-
2	ST	M	30	≥ 30.00	-	-	-
3	SA	F	46	25.52	Sap1<br>Sap2	100%<br>100%	ST2<br>ST2
4	RK	M	34	≥ 30.00	-	-	-
5	DK	F	26	≥ 30.00	-	-	-
6	GM	M	26	≥ 30.00	-	-	-
7	AMa	F	27	19.43	Amap1<br>Amap2	99.90%<br>100%	ST3<br>ST3
8	AD	M	30	≥ 30.00	-	-	-
9	PC	M	26	21.29	-	-	-
10	VT	F	38	20.10	Vtp5<br>Vtp6<br>Vtp7<br>Vtp8	100%<br>100%<br>100%<br>100%	ST4<br>ST4<br>ST4<br>ST4
11	DM	M	50	≥ 30.00	-	-	-
12	RC	M	30	19.08	Rcp22	100%	ST5
13	GR	M	50	18.00	-	-	-
14	AMu	F	40	22.00	Amup9	99.90%	ST6
15	GG	M	33	19.00	-	-	-
16	MC	M	27	26.00	-	-	-

Ct, threshold cycles. Subjects with fecal samples with Ct ≥ 30.00 were considered to have a low count of target bacterial cells and therefore excluded from the isolation procedure.

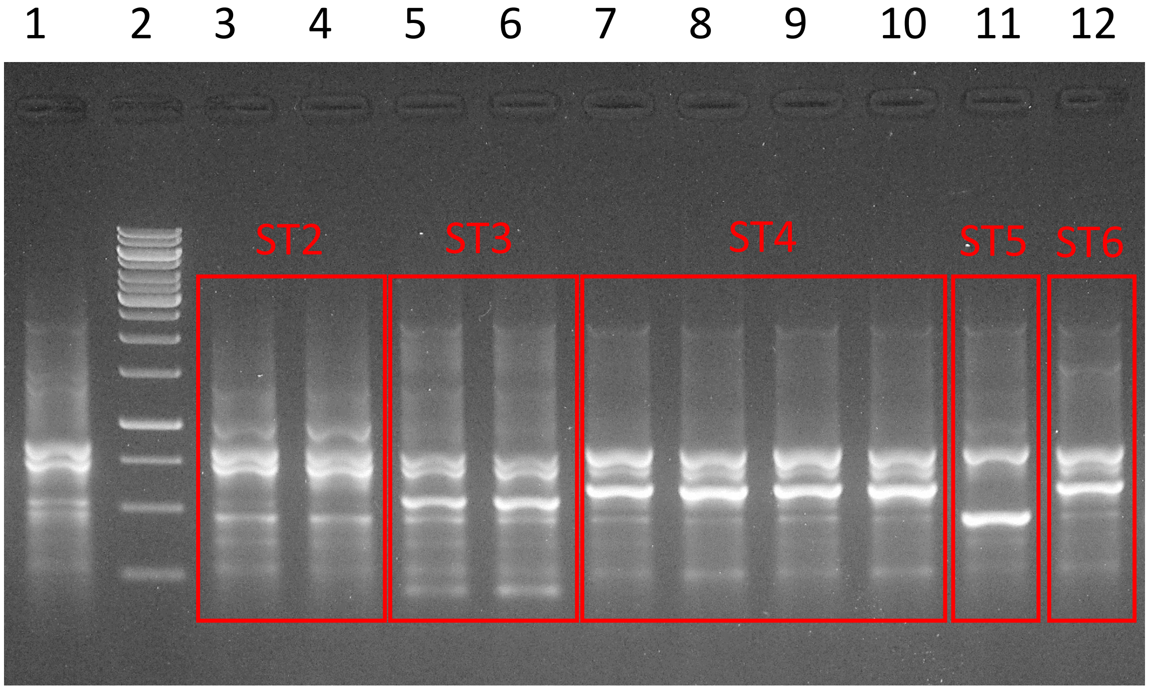

Figure S1. BoxA1 RAPD profiles of *A. muciniphila* strains. Lane 1, *A. muciniphila* DSM 22959^{T}; Lane 2, 1 kb DNA ladder (ThermoFisher); Lanes 3, *A. muciniphila* Sap1; Lanes 4, *A. muciniphila* Sap2; Lanes 5, *A. muciniphila* Amap1; Lanes 6, *A. muciniphila* Amap2; Lanes 7, *A. muciniphila* Vtp5; Lanes 8, *A. muciniphila* Vtp6; Lanes 9, *A. muciniphila* Vtp7; Lanes 10, *A. muciniphila* Vtp8; Lanes 11, *A. muciniphila* Rcp22; Lanes 12, *A. muciniphila* Amup9. BoxA1 RAPD subtypes are boxed in red.

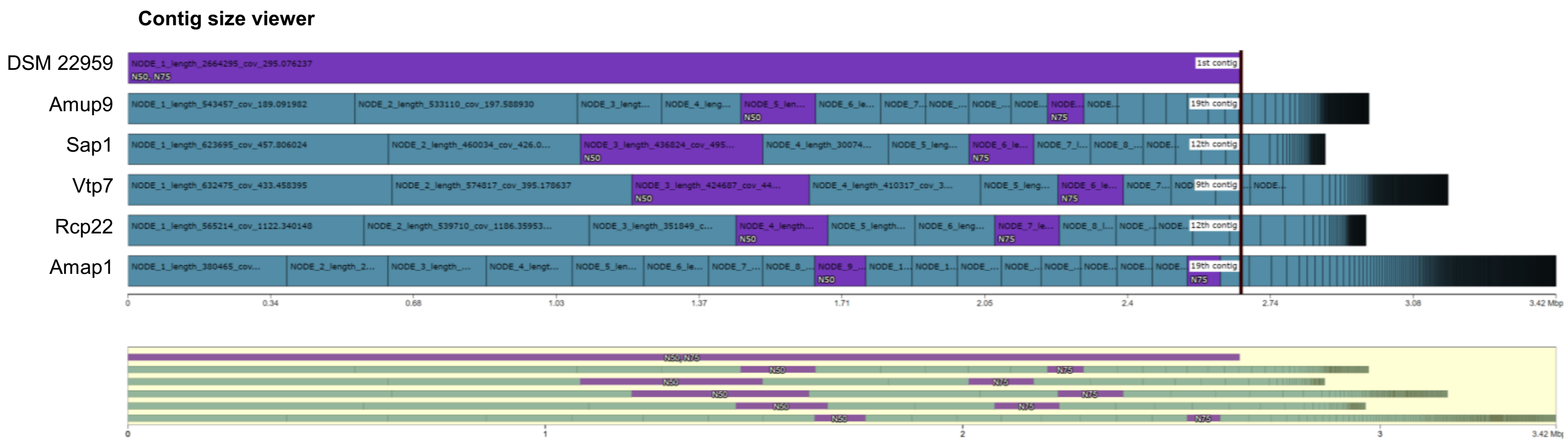

Figure S2. *A. muciniphila* genomes contig size visualization. Contigs for each genome are ordered from longest to shortest. N50 and N75 are also shown. Genomic data were cleaned from contigs < 500 bp (shown after the black line).

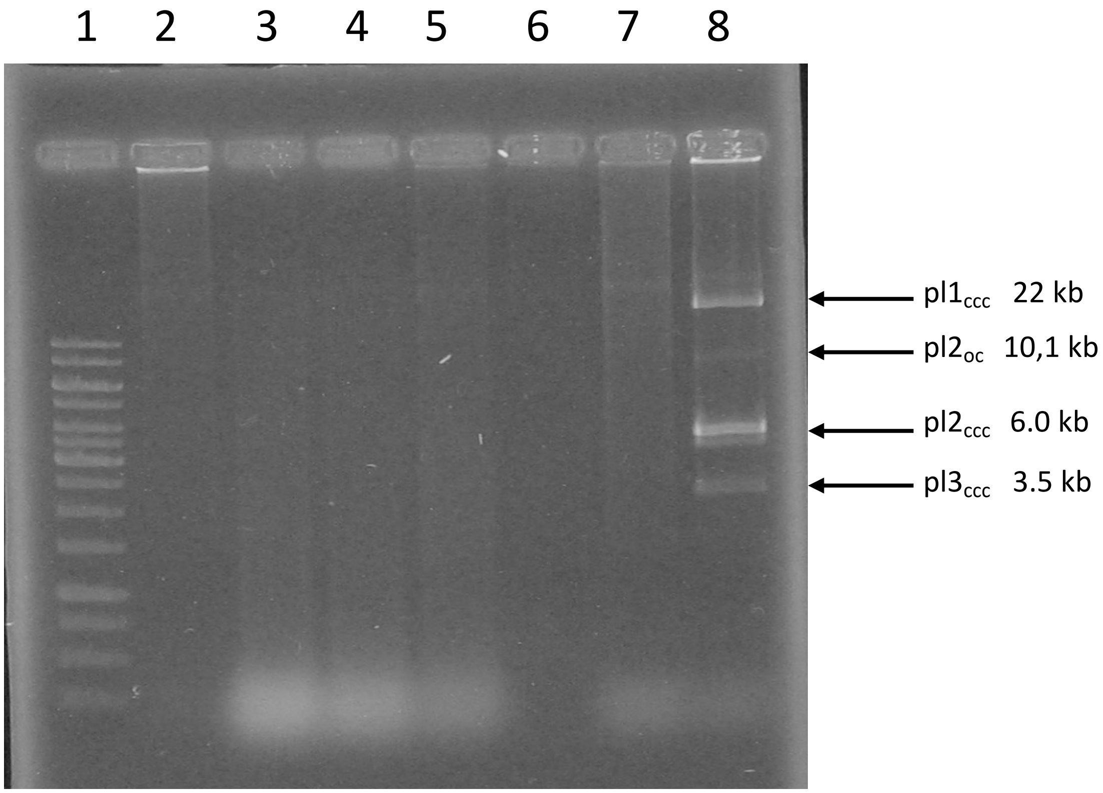

Figure S3. Agarose gel electrophoresis of plasmid DNA extraction from *A. muciniphila* strains. Lane 1, linear 1 kb DNA ladder (ThermoFisher). Plasmid extraction from: lane 2, *A. muciniphila* DSM 22959^{T}; lane 3, Sap1; lane 4, Amap1; lane 5, Vtp7; lane 6, Rcp22; lane 7, Amup9; and lane 8, *Lactobacillus helveticus* ATCC 15009^{T} [S1].

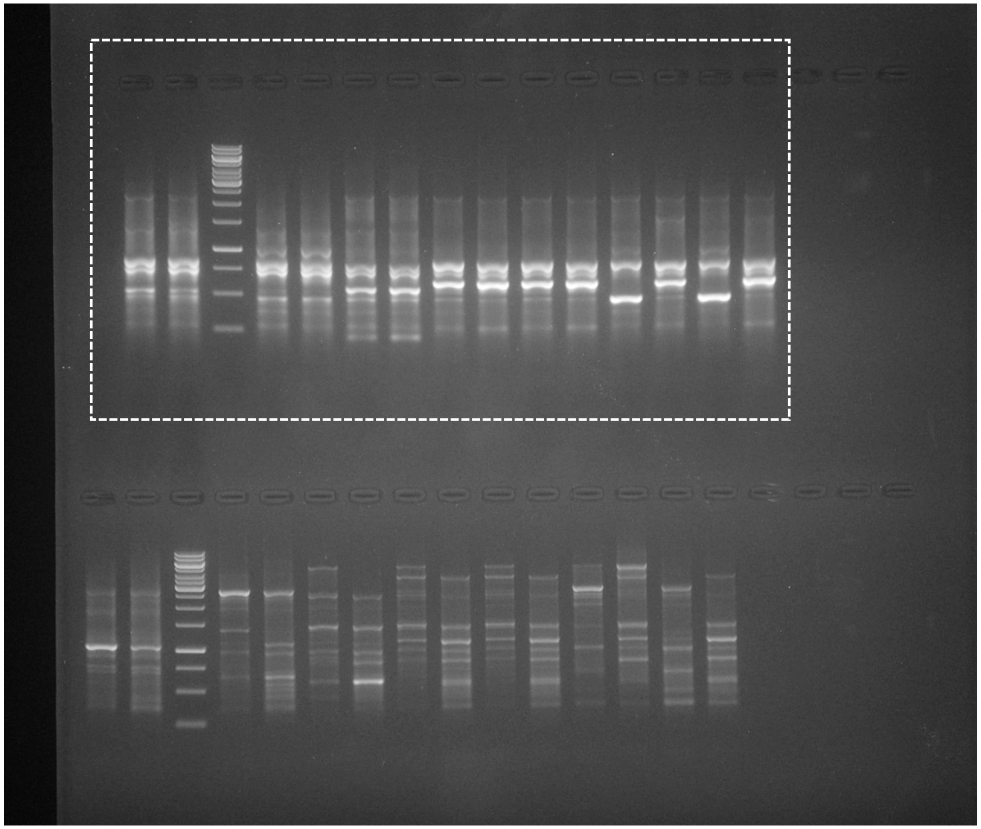

Full-length image of gels used in Figure S1. The dotted gate identifies the cropped area used in Figure S1.

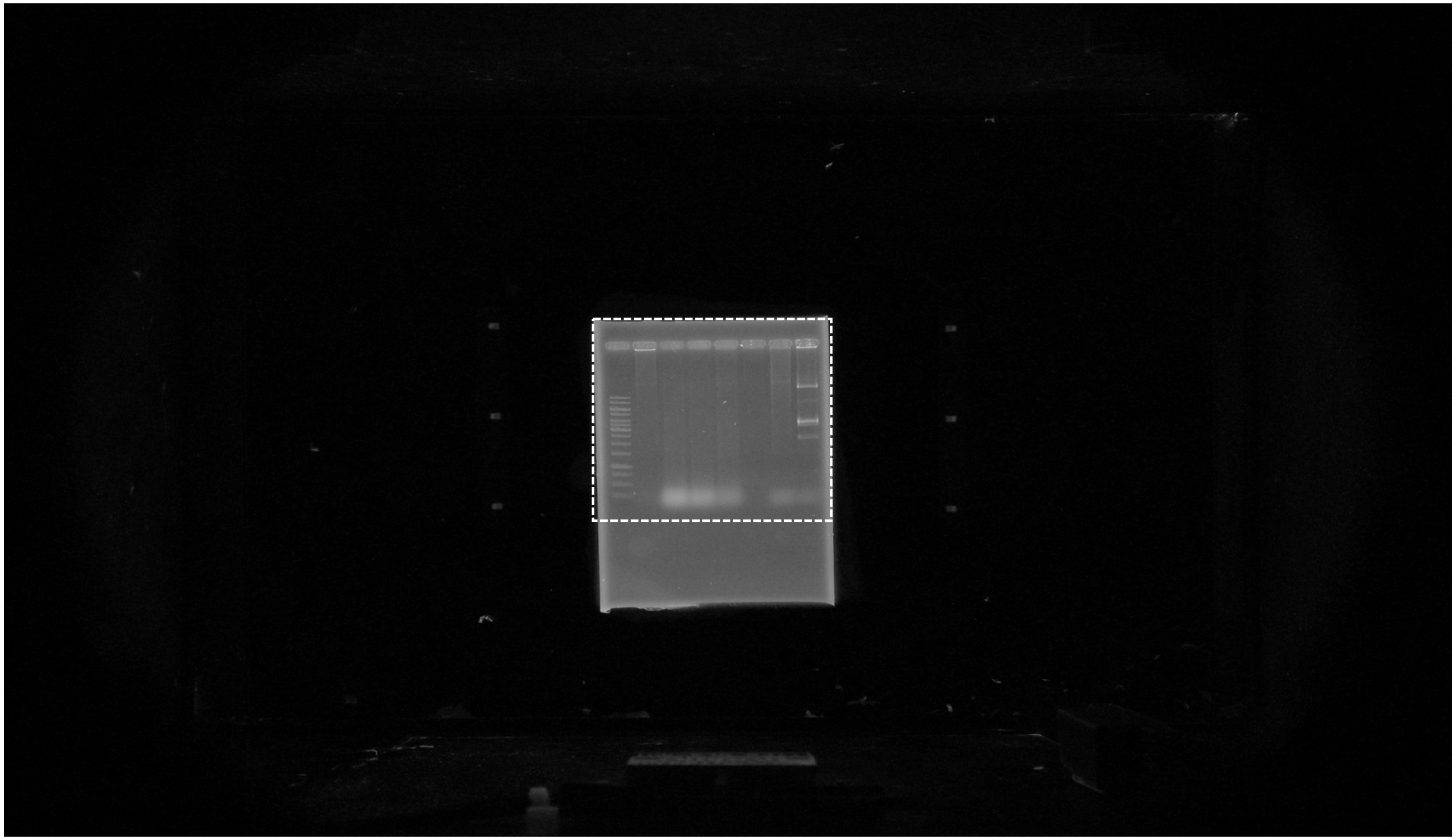

Full-length image of gels used in Figure S3. The dotted gate identifies the cropped area used in Figure S1.

References

S1 Giraffa, G., De Vecchi, P., Rossi, P., Nicastro, G., Fortina, M.G. 1998. Genotypic heterogeneity among *Lactobacillus helveticus* strains isolated from natural cheese starters. J Appl Microbiol 85:411-416.
